# Response to Replication Stress and Maintenance of Genome Stability by WRN, the Werner Syndrome Protein

**DOI:** 10.3390/ijms25158300

**Published:** 2024-07-30

**Authors:** David K. Orren, Amrita Machwe

**Affiliations:** 1 Department of Toxicology and Cancer Biology, University of Kentucky College of Medicine, Lexington, KY 40536, USA; amach0@uky.edu; 2 Markey Cancer Center, University of Kentucky, Lexington, KY 40506, USA

**Keywords:** Werner syndrome, RecQ helicases, genome instability, DNA repair, homologous recombination, replication stress, telomere maintenance

## Abstract

Werner syndrome (WS) is an autosomal recessive disease caused by loss of function of WRN. WS is a segmental progeroid disease and shows early onset or increased frequency of many characteristics of normal aging. WRN possesses helicase, annealing, strand exchange, and exonuclease activities and acts on a variety of DNA substrates, even complex replication and recombination intermediates. Here, we review the genetics, biochemistry, and probably physiological functions of the WRN protein. Although its precise role is unclear, evidence suggests WRN plays a role in pathways that respond to replication stress and maintain genome stability particularly in telomeric regions.

## 1. Introduction

Due to their ability to separate the strands of double-stranded DNA and RNA, helicases are common enzymes in prokaryotic and eukaryotic organisms. They participate in a variety of nucleic acid metabolic transactions including the central processes of transcription and DNA replication as well as recombination and repair pathways that maintain the stability of the genome. The human genome encodes 95 proteins with documented or predicted helicase activity [1], attesting to the importance of these enzymes for proper molecular and cellular function. This review focuses on a very interesting helicase WRN that is deficient in the segmental progeroid disease Werner syndrome (WS). Werner syndrome is a rare autosomal recessive disease that demonstrates early onset and/or higher frequency of certain phenotypes normally associated with human aging, including graying and loss of hair, cataracts, atherosclerosis, osteoporosis, and increased cancers and diabetes mellitus type II [2]. Investigation into the function of the WRN helicase began in earnest after the gene involved in WS was sequenced in 1996 by the Schellenberg and Martin labs at the University of Washington [3]. To researchers interested in connections between DNA damage and aging, this finding was intriguing, as the *WRN* gene sequence revealed that the encoded protein product likely played a role in DNA metabolism. Therefore, initial identification of *WRN* seemed to link a shortfall in DNA metabolic capacity to the early onset and/or increased frequency of the age-related features that are manifested in WS and substantially strengthened a potential causal relationship between genomic instability and other age-related conditions as well as cancer. Although this finding has precipitated a large body of research and literature on WRN, its precise function remains somewhat elusive to this day. Here, we review this literature and the current state of knowledge about the roles of WRN in DNA metabolism. We apologize in advance to those investigators whose important research is not covered in this work.

## 2. *WRN* Genetics

**Figure 1:**
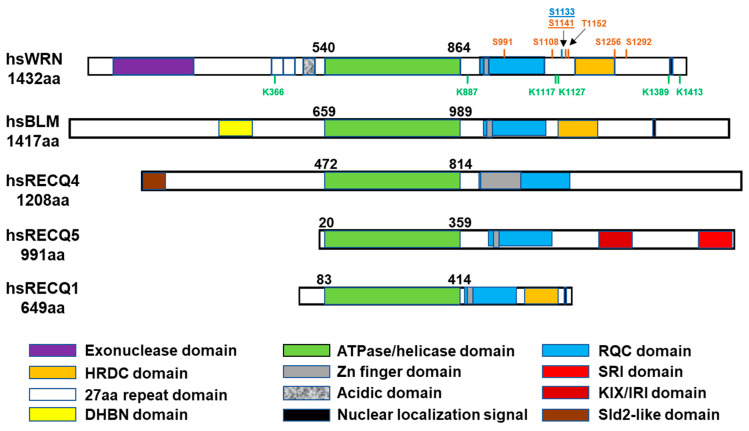
Domain Structure of WRN and Other Members of the Human RECQ family. The domains in the proteins are depicted according to the colors provided. The N-terminal and C-terminal bounds of the ATPase/helicase are indicated by black numbers. Putative ATR/ATM and CDK1 phosphorylation sites are indicated in orange and blue above WRN; putative acetylation sites are indicated in green below WRN. RQC = RecQ-conserved; SRI = Set2-Rpb21 interaction; KIX/IRI = interacting with RNA pol II complexes; DHBN = dimerization helical bundle N-terminal; Sld2-like = region with homology to yeast protein Sld2.

The gene deficient in Werner syndrome was identified by positional cloning to be on chromosome 8 in 1996 and designated *WRN* [3]. The cytogenetic locus of the *WRN* gene is 8p12; its molecular location is NC_000008.11 (31,033,749–31,176,138). Encompassing about 150 kb total, the gene contains 35 exons, although exon 1 produces only 5′-UTR sequences and not any of the protein. Sequencing of the *WRN* gene revealed a predicted ATPase and helicase domain (encoded by exons 14–21) in the central region of the encoded protein, with homology that placed it in the smaller subfamily of RecQ helicases, named for the prototype RecQ protein identified in *E. coli* (Figure 1). While only one RecQ helicase is present in bacterial genomes, yeast has at least two RecQ proteins (Sgs1 and Hrq1 in *S. cerevisiae*, Rqh1 and Hrq1 in *S. pombe*) [4]. Higher eukaryotes have acquired even more RecQ homologs, probably as an adaptation to deal with the increased size and complexity of their genomes. Humans have five RecQ helicases (Figure 1), specifically RECQL (RECQ1), BLM (RECQ2), WRN (RECQ3), RECQ4, and RECQ5. Loss of BLM function causes Bloom syndrome (BS) [5], while loss of or reduced RECQ4 function is associated with Rothmund–Thomson (RTS), Baller–Gerold, and RAPADILINO syndromes [6,7,8]. Recently, germline alteration of RECQL1 has been linked to RECON syndrome, which also shows a genomic instability phenotype [9]. RECQ5 defects have not yet been linked to human disease. Evidence strongly suggests that BLM and RECQ4 are the functional homologs of the yeast Sgs1/Rqh1 and Hrq1 proteins, respectively, and that WRN arose later in eukaryotic evolution. Although chromosomal abnormalities are increased in cells derived from each of these diseases, WS, BS, RTS/RAPADILINO, and RECON are different syndromes with distinct characteristics, suggesting at least some non-overlapping functions.

Further examination of the *WRN* gene sequence uncovered sequence motifs in the N-terminal region (exons 4–7) of the protein previously associated with nuclease activity [10]; these are unique in WRN (and its orthologs) and not found in other human RecQ helicases (Figure 1). However, less conserved domains are shared between some of the human RecQ helicases, including a RecQ-conserved (RQC) domain (exons 22–25) just downstream from the helicase domain found in WRN, BLM, RECQL, and RECQ5 and a helicase and RNase D-conserved (HRDC) domain (exons 30–31) found further towards the C-terminus in WRN, BLM, and some other RecQ family members (Figure 1). *WRN* also encodes a highly acidic region and a 27-amino-acid duplication between its nuclease and helicase domains (Figure 1). We will review in detail below what is known about the helicase function of WRN and its other domains.

At least 87 different *WRN* gene mutations have been identified in WS patients [11,12,13], which can be visualized at the International Registry of Werner Syndrome website (available online: https://dlmp.uw.edu/research-center/werner/registry) (accessed on 5 July 2024). Curiously, the vast majority of these are nonsense, frameshift, splice-site mutations that either prematurely truncate or otherwise drastically alter the encoded WRN protein. It has been suggested that these truncating mutations result in complete loss of function, since they delete the C-terminal nuclear localization sequence and thus prevent entry of any aberrant protein into the nucleus [14]; however, the nonsense-mediated RNA decay process also likely plays a role, since truncated mRNAs and their protein products are generally not detectable in cells with these mutations [15]. Interestingly, five WRN missense mutations do occur in WS patients, two of which (G574R and R637W) lie within the conserved helicase domain [11]. Two pathogenic missense mutations, K125N and K135E, occur within the exonuclease domain, but both appear to cause structural changes that compromise protein stability [16].

Because of the relationship between defective WRN function and aging phenotypes, it is reasonable to think that some *WRN* gene variants might modify the appearance or onset of specific age-related conditions without resulting in the drastic accelerated aging phenotypes of WS. In this regard, sequencing of *WRN* in normal individuals has revealed a number of potentially interesting single nucleotide variants, including V114I (rs2230009), T172P (rs777437332), R834C (rs3087425), L1074F (rs1800392), and C1367R (rs1346044). Notably, V114I, L1074F, and C1367R polymorphisms have been tentatively linked to changes in cholesterol metabolism, cardiovascular disease, and cancer susceptibility, respectively [17,18,19,20,21,22]. However, much more investigation is needed to determine the influence of these and other *WRN* gene polymorphisms on longevity and the appearance and frequency of age-related problems.

## 3. WS Clinical Symptoms

The clinical features of WS have been reviewed elsewhere [2,23] and will be covered only superficially here. WS patients are normal at birth and show no early developmental abnormalities, as opposed to Hutchinson–Guilford progeria and most other segmental progeroid syndromes. This relatively normal period of early development has led researchers to believe that WS may be an acceleration of certain normal aging phenotypes. The first manifestation of being born with two non-functional alleles of *WRN* is lack of an adolescent growth spurt. Skin atrophy and loss of subcutaneous fat appear in WS patients in their twenties, as does loss and graying of hair. Cataracts, a cardinal symptom of WS, appear in their twenties and thirties, occur in essentially all cases, and are mostly observed in both eyes. Other features associated with normal aging begin to appear in their thirties, including osteoporosis, atherosclerosis, gonadal atrophy, and an increased frequency of cancers. There is a higher percentage of tumors of mesenchymal origin, including sarcomas and osteosarcomas, than in the normal population. Type II diabetes mellitus is also observed in a majority of WS cases. Death occurs (by the median age of 54 years) because of heart attacks due to advanced atherosclerosis or cancer. Other features not usually associated with normal aging are short stature, unique “beaked” facial appearance, a high-pitched voice, thin limbs, and flat feet. The different mutations that cause WS appear to lead to the same clinical phenotypes with similar progression, consistent with loss of complete function of the WRN protein; however, it is still possible that certain mutations may have subtle effects on the appearance of particular features.

While increased cancers associated with WS is almost assuredly caused by the elevated levels of genomic instability related to the direct loss of WRN function (see below), the exact cause of other WS phenotypes remains unknown. One can speculate that some of the WS features may be related to limited proliferative capacity because of increased cell death or senescence that occurs as a consequence of DNA and telomeric metabolic defects. Some features of normal aging have been attributed to the accumulation of senescent cells in tissues that produce a senescence-associated secretory phenotype (SASP). Some of the phenotypes of WS may be due to earlier onset of this pro-inflammatory SASP in certain tissues.

## 4. Properties of the WRN Protein

### 4.1. Structural Domains 

WRN is a protein of 1432 amino acids with multiple structural and functional domains (Figure 1). From the N-terminus to C-terminus, these include its nuclease domain (aa 38–230), ATPase domain (aa 540–864), Zn finger (aa 935–946), RQC domain (aa 956–1064), HRDC domain (aa 1142–1235), and nuclear localization signal (aa 1370–1375) [14,24]. The crystal structure of the WRN nuclease domain [25,26] indicates that the catalytic site involves the binding of two metal (Mg^2+^ or Mn^2+^ or Zn^2+^) ions by two key acidic residues, E94 and D95. Mutation of either one of these residues abolishes WRN’s exonuclease activity [27]. WRN’s exonuclease activity is stronger when Mn^2+^ is present as the metal cofactor [25,26]. 

The so-called helicase domain of WRN and other RecQ homologs is actually an ATPase domain that is composed of two subdomains (1A and 2A) identified originally in *E. coli* RecA but present in many other ATPases and helicases. ATP binds in the cleft between the subdomains and, upon hydrolysis, releases energy often used in macromolecular movement (motor function). In WRN and many other helicases, this motor function drives DNA unwinding, even though the primary DNA-binding motifs may lie outside of this domain (see below). In agreement, the isolated ATPase domain of WRN does not efficiently bind DNA or catalyze unwinding [28,29]. Non-conservative mutations at residues in the eight highly conserved sequence motifs in this domain in WRN would be expected to abolish or severely reduce ATPase and thus DNA unwinding activity. This has been shown to be the case for WRN-K577M in motif 1A and (rare polymorphism) WRN-R834C in motif VI [30,31]. The Zn finger sequence in WRN, BLM, and some other RecQ helicases is immediately C-terminal to the ATPase domain. The crystal structures of *E. coli* RecQ [32] and human BLM [33] and RECQ1 [34] indicate that, in three-dimensional space, this Zn finger domain lies up against the ATPase subdomain 2A, and this presumably is the case for WRN as well. These structures essentially show that these Zn finger domains can be considered an accessory module to the ATPase domain present in many RecQ family members.

The RQC domain lies just downstream from the ATPase and Zn finger regions in the primary sequence of WRN, BLM, and other RecQ helicases, and evidence indicates that it also sits close to them in three-dimensional space [33,35,36]. The RQC domain folds into a winged helix (similar to helix-turn-helix motifs) and appears to serve as a major contributor to WRN’s DNA-binding capabilities, as mutations in key residues dramatically decrease DNA-binding affinity and helicase activity [35,37]. Importantly, the structure of the RQC domains of WRN and BLM may act to restrict productive binding to DNA substrates containing ends, and there are β-wing hairpin substructures within these domains that mediate strand separation during unwinding [24,33,35,38]. Thus, the RQC domain probably positions appropriate DNA substrates in such a way that the motor or translocation activity of the ATPase domain can be used to drive strand separation and DNA unwinding. Together, the ATPase, Zn finger, and RQC domains can then be thought of as the helicase “superdomain” of WRN, BLM, and some other RecQ family members.

The HRDC domain, present in WRN and other human RecQ helicases, may also contribute to DNA binding, although existing evidence is contradictory. While the isolated HRDC domain of BLM ortholog Sgs1 of *S. cerevisiae* was shown to bind DNA weakly [39], neither of the isolated HRDC domains from human BLM or WRN could detectably bind DNA in other studies [40,41]. In contrast, some studies indicate that the HRDC domains of WRN and BLM have roles in binding DNA substrates [29,42,43,44,45]. An excellent review of the structural properties of WRN and BLM suggests that their HRDC domains are more likely to mediate key protein–protein interactions than be involved in DNA binding [24]. A more recent structural paper on BLM suggests yet another alternative—that its HRDC domain may have some role in regulating the ATPase/helicase catalytic cycle [36]. Overall, the existing evidence indicates that the HRDC domain has a very modest influence on the core ATPase and helicase activities of WRN and BLM, particularly on the relatively simple substrates used in these structural studies, but do not rule out its role in modulation of activity on the more complex DNA intermediates that appear to be the likely physiological targets of these enzymes. Regardless, the precise function of the HRDC domain in WRN remains to be elucidated.

The WRN nuclear localization signal (NLS) lies near its C-terminus between aa1370 and aa1375 [14,46]. As mentioned above, the vast majority of mutations in WS patients result in truncation of WRN prior to this NLS; thus, any protein produced from these mutants would be unlikely to enter the nucleus and likely result in a complete loss of function. The paucity of missense mutations in WS patients that impact catalytic or functional residues might suggest certain mutant WRN proteins that maintain structural integrity, partial functionality, and nuclear targeting might have highly deleterious dominant-negative effects, precluding survival to term. This notion is supported by some studies in which WRN helicase and exonuclease mutants were ectopically expressed in cell lines [47,48,49,50]. Additionally, a nucleolar localization signal further towards the C-terminus (aa1403–1406) of WRN was also identified [46]. This sequence seems to confer the observed enrichment of WRN in this organelle, at least under unperturbed conditions [51,52].

### 4.2. DNA Binding

As might be expected of a helicase with a putative role in maintaining genome stability, WRN is a DNA-binding protein. Although originally shown to bind DNA structures with single-stranded character more strongly than fully duplex structures [53], more in-depth experiments have revealed that WRN prefers to bind to certain DNA structural features and has particularly high affinity for complex three- and four-stranded replication and recombination intermediates. Specifically, WRN has enhanced affinity to two-, three-, and four-stranded forks, bubbles, D-loops, and Holliday junctions [54,55,56,57]. It is notable that, on many of these substrates, WRN can bind near the junctions between single- and double-stranded DNA or precisely around the Holliday junction [54,56,58,59]. Although a systematic comparison of WRN binding to structurally distinct substrates has not been published, experiments performed in our lab suggest that WRN has highest affinity to three- and four-stranded fork, D-loop, and Holliday junction structures (Orren and colleagues, unpublished observations). While early studies did not reveal any DNA sequence specificity for WRN binding, experiments from our lab indicate that the presence of telomeric G-rich sequences at a specific site in a particular structural context (a D-loop) enhanced binding to and activity of WRN on this structure [54]. This may underlie a preferential role for WRN in telomere maintenance (see below).

### 4.3. ATPase, Helicase, and Branch Migration Activities

WRN and other RecQ family members belong to the SF2 helicase superfamily. The ATPase/helicase domain of WRN spans amino acids 540–864. This includes the seven classic ATPase and helicase motifs (identified as I, Ia, II, III, IV, V, and VI) plus a motif 0 (motif Q) upstream of the other motifs (amino acids 540–561 in WRN) present in RecQ family members [60]. The ATPase activity of WRN is dependent on Mg^2+^ and stimulated by single-stranded DNA to a much greater degree than by double-stranded DNA [29,52]. As is typically the case for helicases, ATPase activity is necessary for WRN’s ability to unwind DNA duplexes; a K577M mutation in motif I disables both ATPase and helicase activities [30]. As for other helicase activities of RecQ family members established thus far, the directionality of WRN’s helicase activity is 3′ to 5′, defined initially on simple duplex substrates with single-stranded regions 3′ to the duplex to be unwound [61]. On these substrates, the ATPase activity of WRN mediates its 3′ to 5′ translocation along single-stranded DNA; upon encountering a duplex region, WRN can then displace (5′ to 3′) the other strand. On its own, WRN is a relatively weak helicase, being able to only unwind short DNA duplexes of less than 42 bp [61]. This may be due, at least in part, to WRN’s annealing activity (see below) that tends to counteract unwinding; indeed, prevention of re-annealing substantially increased unwinding strength for not only WRN but also BLM [62]. Since the DNA-binding affinity of WRN appears to be conferred primarily by regions outside the helicase domain, in biochemical assays this positively influences observed unwinding. In our experience, DNA substrates that are bound better by WRN require less protein to unwind duplexes of comparable length. For example, a forked substrate with a fixed duplex region would be unwound more readily by WRN than a 3-overhang substrate with an identical duplex region [63,64]. Additional studies indicate that even more complex structures including three- and four-stranded forks, static D-loops, and Holliday junctions are also readily unwound by WRN [57,58,64,65]. In contrast to the translocation mechanism mentioned above, WRN unwinding on these substrates can be initiated by binding to their structural features (such as junctions between single- and double-stranded DNA) and not necessarily to a significant stretch of single-stranded DNA.

G-quadruplexes are secondary DNA structures mediated by Hoogsteen base pairing that can form from certain G-rich single-stranded regions, such as those found in telomeres. Due to its putative role in telomere maintenance (see below), the ability of WRN to unwind (or more appropriately, disrupt) G-quadruplexes has been examined. The results of those studies indicate that WRN can indeed unfold these structures in vitro in a reaction that depends on ATP hydrolysis [64,66]. However, a short stretch of single-stranded DNA adjacent (optimally 3′) to the G-quadruplex structure appears to be needed for this activity, suggesting that the mode of action is the initial binding of WRN to this single-stranded region followed by its ATPase-driven 3′ to 5′ translocation to disrupt these G-quadruplexes. WRN’s ability to bind to and unfold G-quadruplexes appears to involve key residues (T1024, L1066, and T1086) in its RQC domain; intriguingly, these residues have no effect on the unwinding of duplex DNA molecules [67]. It is noteworthy that BLM and other helicases [42,64,68,69,70] have also been shown to unfold G-quadruplexes, including the non-RecQ helicases Pif1/PIF1 [71] and FANC-J [72,73], which seem to have the key role in dealing with these structures within the *S. cerevisiae* and human genomes, respectively [72,74]. FANCJ may cooperate with WRN (and/or BLM) to facilitate unfolding of G-quadruplexes [75,76]. 

The weak helicase activity of WRN is stimulated by its interaction with replication protein A (RPA) [61,77], the heterotrimeric single-stranded DNA-binding protein that participates in DNA replication, recombination, and repair pathways. The length of duplex DNA that can be unwound by WRN is markedly stimulated by human RPA, and its stimulatory effect is significantly stronger than single-stranded binding proteins from bacteria or T4 phages [61,77], indicating that this stimulation is not only due to trapping of the unwound strands by RPA but also involves the direct interaction between WRN and RPA. This direct interaction was subsequently mapped to a region (aa 168–308) on the 70 kDa subunit of RPA and primarily to a region in WRN between the exonuclease and helicase domains [78,79]. Notably, RPA can also specifically stimulate unwinding by the other human RecQ helicases BLM, RECQ1, and RECQ5β [80,81,82].

As mentioned above, WRN has high affinity for D-loop/three-way junction and four-stranded Holliday junctions (HJs) that reflect key recombination intermediates [54,57,64,83]. When such substrates lack internal homology, WRN can mediate unwinding of one or more strands of these complex three-way junction and HJ structures. However, during physiologically relevant recombination processes, such structures have a high degree of homology, meaning they can undergo branch migration. Notably, when three-way junctions and HJ substrates are designed in a way that branch migration can be assessed, WRN can use its inherent ATPase and helicase activities to catalyze this process [84,85,86]. Importantly, branch migration seems to be a property conserved in other prokaryotic and eukaryotic RecQ proteins [87,88,89]. Thus, the high DNA-binding affinity and branch migration activity on recombination structures support the concept that WRN and some other RecQ enzymes act to process these intermediates. In agreement, structural models suggest that WRN and BLM interact with strand invasion intermediates/D-loops and Holliday junctions in a manner that would facilitate branch migration of these structures [24].

### 4.4. Annealing Activity

As a RecQ helicase, the ability of WRN to separate DNA strands in a manner dependent on ATP hydrolysis was predictable and readily demonstrated on a series of duplex DNA structures. Unexpectedly, our lab discovered that WRN had the ability to dramatically accelerate annealing of complementary single-stranded DNA [55]. Specifically, the rate of pairing of completely complementary 80 nt oligomers at 12.5 pM each was increased by over 250-fold, compared to the rate of spontaneous annealing [55]. WRN’s annealing activity was observed in other DNA structural contexts, including pairing of a third (partly complementary) DNA strand to an existing forked DNA structure. While this action would seemingly counteract DNA unwinding, it could be envisioned that annealing and helicase activities might instead be coordinated to carry out more complex DNA transactions that typically occur in recombination pathways. Indeed, WRN-mediated catalysis of branch migration, strand exchange, and replication fork regression has been documented in biochemical assays [55,84,85,86,90], all of which could be facilitated by coordination between its unwinding and annealing activities. Importantly, annealing activity seems to be a property of many RecQ helicases, including the human BLM, RECQ1, and RECQ5β proteins [55,82,91,92]. This supports the concept that those RecQ family members with annealing activity may have important functions in recombination processes.

### 4.5. Exonuclease Activity

As mentioned above, WRN is the lone human RecQ family member to possess domains homologous with known nucleases [10]. Shortly thereafter, the presence of a 3′ to 5′ exonuclease activity in WRN was observed by several research groups [27,61,93,94]. The N-terminal region (amino acids 1–369) containing only this domain retains this activity, and an E84A mutation at a conserved catalytic residue abolishes it [27], confirming that this conserved domain is responsible for the 3′ to 5′ exonuclease function. An early observation of a 5′ to 3′ exonuclease activity associated with WRN [95] has not been reproduced and was likely related to a contaminant that co-purifies with WRN during His-tag affinity chromatography [53]. While initially observed by the stepwise degradation of a 5′-radiolabeled duplex substrate with a 3′ overhang [27], WRN’s exonuclease activity is much more robust in other DNA structural contexts, including on long single-stranded oligomers, bubble-containing substrates, and the leading daughter strand of artificial replication fork substrates [56,86,96]. In these contexts, it is clear that DNA-binding domains outside of the exonuclease domain contribute substantially to this increased activity, as the WRN nuclease fragment mentioned above loses such specificity [56]. This has led to some investigation and speculation that the exonuclease function of WRN is somehow coordinated with its other activities. 

WRN’s 3′ to 5′ exonuclease activity is easily identified on undamaged substrates ending with standard 3′-OH termini. In contrast, this activity is blocked by the presence of certain damaged nucleotides [97] as well as abnormal 3′ end groups including 3′ phosphates and 3′ phosphoglycolates [98] that are often produced during DNA breakage by chemical and physical agents. The exonuclease activity of WRN also appears to be blocked or very strongly inhibited by artificial phosphorothioate bonds that can be incorporated between nucleotides during commercial synthesis (Orren and colleagues, unpublished observations). Although these findings argue against roles for WRN in DNA damage processing including end processing of double-strand breaks, these restrictions on its exonuclease activity can be exploited to prevent unwanted degradation of substrates or of specific 3′ ends in the design of specific biochemical assays. 

The exonuclease activity of WRN can be influenced by interactions with other proteins. Ku, the heterodimeric complex that encircles DNA ends and initiates the non-homologous end joining (NHEJ) pathway for double-strand break repair markedly stimulates the exonuclease activity of WRN, allowing degradation even through DNA regions containing damaged nucleotides (including 8-hydroxyguanine lesions) that block WRN alone [93,99,100]. Another key NHEJ factor, DNA-PKcs, also seems to influence WRN activity. In one study [101], DNA-PKcs alone did not influence WRN but actually inhibited the Ku-mediated stimulation of WRN exonuclease activity; this inhibition required the kinase activity of DNA-PKcs and was reversed by phosphatase treatment, suggesting phosphorylation of WRN by DNA-PK and a possible mode of regulation. In another study [102], DNA-PKcs inhibited WRN helicase activity which was reversed by Ku and independent of DNA-PKcs phosphorylation of WRN. Telomeric factors also appear to be able to regulate WRN exonuclease activity. WRN-mediated exonucleolytic degradation of substrates specifically containing telomeric duplex regions is stimulated by TRF2 but not by TRF1, even though both of these telomeric factors readily bind these substrates [103], suggesting that TRF2 recruits WRN to telomeric DNA. In another study [65], TRF1 and TRF2 modulate the exonuclease activity of WRN on D-loop structures with telomeric sequences.

## 5. Likely Physiological Roles

**Figure 2:**
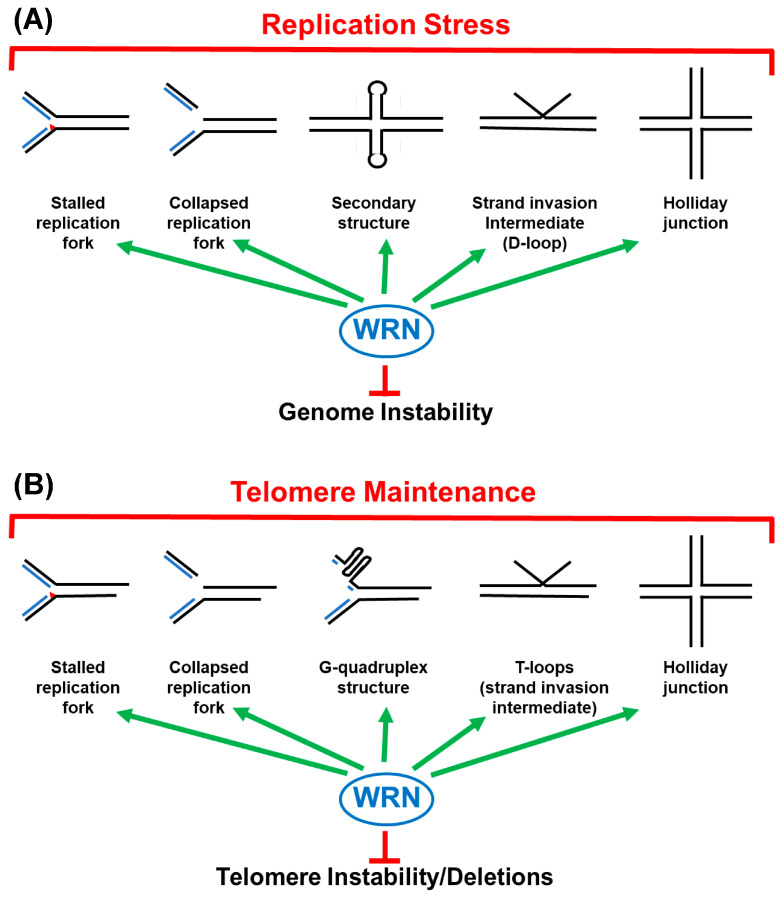
**Various activities of WRN in combating replication stress and in telomere maintenance**. (**A**) WRN, with its inherent helicase, strand exchange, branch migration, and exonuclease activities, has been shown to have a preference for processing the structures that cause or are induced by replication stress. Loss of WRN function results in genome instability. (**B**) Because telomeres are formed with repeating GGGTTA/CCCAAT sequences, they are naturally difficult to replicate through and can form structures such as G-quadruplexes and T-loops. The replication fork may stall and collapse in telomeres because of replication stress, leading to WRN-mediated action to rescue telomeric replication and/or promote recombinational repair. Loss of WRN results in telomere instability, primarily stochastic telomeric deletions.

While biochemical and enzymatic characterization of WRN has provided valuable insights, elucidation of its precise physiological roles with cells and tissues has proven to be more challenging. Research on cells derived from WS patients predated discovery of the *WRN* gene and has continued since, and genetic manipulation techniques have facilitated studies of cells and animal models with reduced or completely abrogated WRN function. As a whole, these studies have revealed molecular and cellular phenotypes that point to WRN’s participation in several metabolic pathways. Importantly, most of the implicated pathways are important for maintaining the stability of the human genome. In broad terms, loss of WRN function directly increases the pace of genomic change in individual cells that appear to result in disparate outcomes. In one readily understood scenario, increased genomic changes (in this case, chromosomal alterations) at the cellular level lead to a higher probability of cell transformation and increased susceptibility to cancer development, as has been demonstrated for many genomic instability syndromes. In another scenario, genomic changes cause increased occurrence of either cell death or cell senescence in cells lacking WRN function. Cumulative cell death and cell senescence over time can reduce the proliferative and regenerative capacity of tissues and contribute to age-related phenotypes. Moreover, accumulation of aberrant senescent cells in tissues is now believed to cause additional problems (via the so-called senescence-associated secretory phenotype) that may contribute to aging (reviewed in [104]). However, it should be kept in mind that these outcomes probably occur at different rates in distinct cell types and tissues, which could be related to the acceleration and/or earlier onset of the particular subset of age-related features observed in WS patients. Most studies suggest that WRN is involved in the response to replication stress (Figure 2A), although more research is needed to understand the connections between the genome maintenance defects caused by WRN loss and at least the non-cancer phenotypes that are associated with WS.

In unperturbed wild-type cells, WRN tends to have either an enriched nucleolar localization or diffuse nuclear localization [14,46,51,52]. However, following treatment with a variety of DNA-damaging agents, WRN relocalizes to discrete nuclear foci containing RPA that appear to correspond to sites of ongoing replication [84,105,106], an event that coincides with acetylation of WRN [106,107,108]. These acetylation events have been suggested to change the DNA-binding properties of WRN and protect it from ubiquitination and degradation [108].

### 5.1. Genome Stability

As alluded to above, cells lacking WRN function show elevated levels of chromosomal instability, a phenotype typical of many RecQ helicase-deficient conditions, indicating that many RecQ family members suppress chromosome rearrangements. The specific chromosomal instability phenotype originally discovered in fibroblasts from WS patients was termed variegated translocation mosaicism [109,110], meaning that clonal populations within the cultures carried the same chromosomal rearrangements. Chromosomal rearrangements were also found in lymphocytes as well as in early outgrowths of skin explants from WS patients [110], strongly suggesting that these chromosomal aberrations were formed in vivo and were a result of WRN deficiency and not an artifact of growth in culture. Later studies showed a variety of chromosomal aberrations including deletions, insertions, and translocations were elevated in WRN-deficient cells and further increased by treatment with certain DNA-damaging agents [111,112]. Taken together, these early studies demonstrated that loss of WRN function leads to increased frequencies of large-scale chromosomal aberrations. These events are known to be the result of misjoining of DNA double-strand breaks (DSBs). Therefore, two broad scenarios can be envisioned. In the first scenario, loss of WRN function directly leads to an increase in the misjoining of DSBs, possibly because of an alteration in a double-strand break repair pathway. In the second scenario, WRN deficiency causes an increased frequency and/or persistence of DSBs that raises the chances of misjoining. Evidence exists supporting both of these scenarios.

When a cell type shows a genome instability or mutator phenotype, a common strategy is to challenge those cells with DNA-damaging agents in an attempt to determine the genome maintenance pathway(s) that are likely to be defective. Thus, WRN-deficient cells have been treated with a variety of genotoxic agents and assayed for increased loss of viability (hypersensitivity) or other relevant endpoints. At best, only a very mild sensitivity of WRN-deficient cells to ionizing radiation has been observed [102,113], which is somewhat surprising due to the aforementioned possibility of a defect in the repair of DSBs that would be induced by this agent. WRN-deficient cells are also not particularly hypersensitive to ultraviolet radiation [114,115]. Of the DNA-damaging compounds surveyed, WRN-deficient cells seemed to be most consistently hypersensitive to agents that produced interstrand crosslinks including cisplatin, mitomycin C, and melphalan [116,117]. Notably, WRN-deficient cells are also hypersensitive to 4-nitroquinoline-1-oxide, some alkylating agents, and topoisomerase I inhibitors such as camptothecin (CPT) [115,116,118,119,120,121,122]. This curious array of hypersensitivities is not consistent with a defect in canonical DNA repair pathways such as alkyltransferase, nucleotide excision repair, base excision repair, mismatch repair, or even double-strand break repair. Instead, one commonality was that the agents to which WRN-deficient cells were hypersensitive all induced at least some chemical or structural alterations that could block or stall replicative DNA polymerases. Importantly, WRN-deficient cells are also hypersensitive to treatment with hydroxyurea (HU) or aphidicolin [121,123,124], compounds that do not cause chemical changes in DNA but instead stall replication by depletion of nucleotides or direct inhibition of replicative DNA polymerases, respectively. Together, these hypersensitivities suggest that WRN is, at least, involved in the cellular response to replication blockage or stalling (see below). In general agreement with this notion, apoptotic cell death in WRN-deficient cells following such treatments appears to occur primarily during the S or G2 phase of the cell cycle [123].

### 5.2. Double-Strand Break Repair

The elevated chromosomal rearrangements observed originally in cells from WS patients raised the possibility of a defect in normal joining of DSBs. Two-ended DSBs, such as those generated by ionizing radiation and oxidative damaging agents, are typically repaired by either non-homologous end joining (NHEJ) or homologous recombination (HR) pathways. NHEJ pathways, responsible for DSB repair in differentiated cells and in the G1 phase of proliferating cells, splice together the two broken ends of duplex DNA, often leading to some loss of sequence (deletion) around the site of the break. In contrast, HR pathways in mitotic cells are only utilized in S and G2 phases, and an intact highly homologous intact duplex (usually the sister chromatid) is used as a template to repair and accurately restore the sequence and structure of the broken duplex. A number of studies have suggested that WRN may play roles in NHEJ and/or HR pathways (see below). By this reasoning, loss of WRN function would lead to persistence of DSBs and/or utilization of more error-prone DSB repair pathways that, in turn, could result in increased cell death or cellular senescence or elevated numbers of chromosome aberrations that are characteristic of WRN-deficient cells.

#### 5.2.1. NHEJ

NHEJ mechanisms involve bridging the two duplex ends, processing of aberrant end groups and flaps, DNA synthesis to fill any resulting gaps, and, finally, ligation. Canonical (or classical) NHEJ (cNHEJ) is the primary NHEJ pathway and requires the heterodimeric Ku factor (Ku), DNA-dependent protein kinase catalytic subunit (DNA-PKcs), end-processing factor Artemis, and XRCC4/DNA ligase IV, as well as DNA polymerases and other end-processing factors. Alternative NHEJ (alt-NHEJ) appears to serve mainly as a backup pathway for both cNHEJ and HR and is much more prone to result in extensive deletions or other chromosomal aberrations. In end-rejoining assays using plasmid vectors, WRN-deficient cells showed more extensive deletions during rejoining than wild-type cells [125], suggesting possible use of alt-NHEJ instead of cNHEJ. As mentioned above, WRN interacts with several key cNHEJ factors, including Ku and DNA-PKcs [93,101,102,126,127]. Ku interacts directly with WRN and strongly stimulates its 3′ to 5′ exonuclease activity, even its ability to degrade through DNA containing certain types of damage including the major oxidative lesion 8-hydroxyguanine [93,100]. However, WRN exonuclease cannot remove aberrant 3′ end groups that are often induced at DSBs, and its interaction with Ku appears not to change this restriction [98]. DNA-PKcs interacts with WRN in a manner that regulates its enzymatic activities, and Ku, DNA-PKcs, and WRN can form stable complexes on DNA ends [101,102]. The finding that WRN-deficient cells are, at best, mildly sensitive to IR strongly suggests that WRN may not be essential for most NHEJ repair events, in contrast to the roles of Ku, DNA-PKcs, and other core NHEJ factors. Perhaps WRN serves as an accessory end-processing factor for a subset of NHEJ reactions, and/or other processing factors can substitute for WRN in its absence.

#### 5.2.2. HR

HR repairs DSBs in mitotic cells via complex pathways in which many of steps are now outlined but not yet completely elucidated. HR pathways are initiated by resection from the 5′ end of a DSB, leaving a 3′ overhang that is the substrate for RAD51-mediated formation of filaments, protein–DNA structures crucial for the subsequent strand invasion, and exchange steps that occur with the intact homologous duplex partner. After DNA polymerases use the homologous template to extend/resynthesize the broken invading strand, the later steps that completely re-establish the broken duplex remain unclear, but resolution of the complex DNA structures must occur. Much of the support for a role for WRN (and other RecQ family members) in HR processes stems from our understanding of the function of the prototypic RecQ protein in the RecFOR pathway in *E. coli* and the biochemical similarities between RecQ family members. Notably, the RecFOR pathway seems preferentially involved in HR pathways responding to single-stranded gaps resulting from unreplicated DNA and/or collapsed replication forks [4]. RecQ has a key role in the RecFOR system of recombination in bacteria [87,88,128,129]. Many other RecQ family members including WRN bind with high affinity to complex three- and four-stranded structures reminiscent of replication and recombination intermediates [54,59,85,86] and readily catalyze branch migration [85,86], an activity that could theoretically facilitate productive HR reactions or reverse non-productive ones. WRN itself has extremely high affinity for strand invasion intermediates and can readily catalyze strand exchange reactions [54,55,90]. The strand annealing properties of WRN and other RecQ family members [55,91,130] seem likely to contribute to their branch migration and strand exchange activities. As mentioned previously, WRN interacts directly with RPA [59,77,131], which has a role in protecting resected 3′ overhangs prior to RAD51 filament formation during HR. WRN also has interactions with HR factors including the MRN complex potentially through direct interaction with its NBS1 subunit [132,133] that first binds to DSBs and directs ATM checkpoint activation and end resection. WRN also interacts with the RAD51, RAD52, and RAD54B proteins that participate in later HR transactions [134,135]. Thus, the properties inherent in WRN and many other RecQ family members suggest that these helicases have evolved to participate in HR processes.

### 5.3. Long-Range Resection

Studies over the last decade have somewhat clarified the resection step of HR and, in the process, promoted a role for certain RecQ helicases. WRN has interactions with several factors known to be involved in HR. Initial resection from the 5′ ends of DSBs is performed by the coordinated action of the end-binding MRN complex and CtIP factor and activated by phosphorylation of the latter by S phase cyclin-cdk kinases; this restricts HR processes to S and G2 phases (reviewed in [136,137]). Subsequently, further “long-range” resection to generate extensive 3′ overhangs sufficient for filament formation is apparently performed by either DNA2- or EXO1-dependent nucleolytic degradation [138,139,140]. Importantly, the DNA2-mediated resection apparently involves RecQ helicases—Sgs1 in *S. cerevisiae* [141,142] and either BLM or WRN in human cells [143,144]. In each case, helicase activity of the relevant RecQ family member was required, presumably to generate the optimal 5′ flap substrate for DNA2-mediated strand cleavage (similar to the situation with FEN1). A study by Pichierri and colleagues [50] strengthened the concept that WRN could act in this long-range resection step. This research identified a conserved CDK1 phosphorylation site in WRN that stabilized MRN localization at DSBs and regulated DNA2-mediated resection, particularly on one-ended DSBs formed by replication fork collapse [50]. The involvement of WRN in HR-mediated repair of replication-related DSBs would be consistent with the slowed/perturbed replication dynamics of WRN-deficient cells, their hypersensitivity to agents that disrupt DNA replication, and recruitment of WRN to replication foci after damaging treatments (see below). However, the physiological complexity is striking, as in cells with both BLM and WRN homologs, these helicases can substitute for one another in long-range resection, and the EXO1-dependent resection pathway also is available; thus, neither WRN nor BLM is essential for HR to occur, although each might be more utilized under specific circumstances. Notably, the exonuclease activity of WRN is apparently not involved in this long-range resection step of HR.

### 5.4. Rescue of Stalled or Collapsed Replication Forks

As mentioned above, WRN-deficient cells seem to be particularly sensitive to agents known to block or stall DNA replication. Research over the last two decades has revealed that replication blockage occurs commonly in dividing cells. Even in cells not exposed to exogenous DNA-damaging agents, replication blockage may be caused by endogenous DNA damage or by difficult-to-replicate regions of the genome that inhibit progression of the DNA replication machinery. Additional evidence suggests that WRN functions in the response to blocked replication forks or to collapse of those replication forks subsequent to blockage. Early experiments showed that replication was somewhat slowed and S phase extended in WS fibroblasts [145,146]. This defect was further elucidated by DNA-combing experiments showing that progression of replication forks in WRN-deficient cells is asymmetric and slower [147,148], suggesting more frequent, extended stalling of replication. In the presence of aphidicolin concentrations that slow replication, loss of WRN helicase (but not exonuclease) activity is associated with increased breakage of certain fragile sites in a manner that appears epistatic to the replication checkpoint factor ATR [124,149]. Notably, in response to treatment with HU or CPT, WRN is also phosphorylated by ATR, which appears to regulate WRN’s role in response to stalled replication [115,150] perhaps in stabilizing stalled forks. A specialized situation that results in fork stalling is conflicts between transcription (and the presence of R loops) and replication. Recently, WRN has been implicated in dealing with conflicts between transcription and replication, preventing genomic instability at these sites in the genome [151]. The response to replication blockage is very complex, and the DNA transactions that may occur at the blocked fork are still not well defined. WRN recruitment to sites of blocked replication suggests that its catalytic activities may act on secondary DNA structures that either block replication or on different intermediates that arise subsequent to fork stalling; several specific roles have been advanced by experimentation.

The single-stranded DNA-binding factor RPA is a key player in many DNA metabolic processes, particularly in replication and recombination pathways. Lagging strand replication, replication fork stalling, and resection steps of HR pathways generate single-stranded DNA initially bound and protected by RPA. Importantly, RPA bound at single-stranded gaps formed upon replication stalling has been demonstrated to be critical in activating the replication checkpoint governed by the ATR kinase [152]. As mentioned above, RPA directly and functionally interacts with WRN and stimulates its unwinding activity [77,131]. Thus, it is very tempting to speculate that this interaction facilitates WRN action at sites of replication blockage or collapsed replication forks, possibly also in coordination with ATR activation. In agreement, binding of RPA to a model replication fork facilitates WRN recruitment and action (fork regression) in vitro, with RPA being displaced by WRN in the process [59]. More research is needed to specify the function of WRN in the responses to replication fork perturbation and how this is coordinated with other factors thought to be involved in these responses including RPA and ATR.

### 5.5. Regression of Replication Forks or Their Re-Establishment 

Upon replication blockage, one proposed scenario is that the four-stranded replication fork can regress, involving re-pairing of the parental strands along with pairing of the nascent daughter strands to form a “chicken foot” structure that essentially is a Holliday junction. Although it has been suggested that such a structure can be formed spontaneously due to forces related to supercoiling ahead of the replication fork, it is striking that WRN and BLM can very efficiently regress model replication fork substrates to yield the chicken foot structure and that this activity is dependent upon their ATPase and helicase activities [86,153]. Furthermore, the 3′ to 5′ exonuclease activity of WRN on the leading daughter strand of such substrates can further facilitate fork regression in certain circumstances [86]. These findings suggest that WRN (or BLM) might assist in regression of blocked replication forks to form this chicken foot intermediate. Importantly, formation of this structure might lead to one of several beneficial outcomes. In one scenario, repairing of a parental strand around a replication-blocking DNA lesion could allow access to and lesion removal by canonical DNA repair pathways. In another scenario, a daughter strand pairing may permit additional DNA synthesis using the nascent lagging strand as a template, with subsequent reversal of the chicken foot back to the replication fork resulting in lesion bypass. In a third scenario, the chicken foot structure becomes a substrate for action by Holliday junction resolvases, creating a DSB that could become a necessary intermediate in replication restart pathways. If replication forks are indeed regressed under certain circumstances, regenerating the standard replication fork structure could be a step to facilitate resumption of normal DNA replication. Importantly, either WRN or BLM can catalyze this DNA transaction on model chicken foot/Holliday junction structures [85]. This reaction involves the ATPase and helicase activities of these proteins and likely occurs through their ability to catalyze branch migration [85]. WRN helicase and exonuclease appear to function during and/or subsequent to fork regression; the former prevents increased DNA breakage and genome rearrangement while the latter inhibits extensive nascent strand degradation and RAD51-mediated restoration of replication [47,49].

### 5.6. Break-Induced Repair of Collapsed Replication Forks

Replication forks are also known to collapse and, in the process, generate DSBs. This can occur directly upon encounter of a single-strand break in one of the parental strands by the replication fork or subsequent to a replication fork-blocking event. Such scenarios are thought to primarily yield a “one-ended” DSB that cannot be repaired by standard NHEJ pathways. Instead, such events appear to induce an HR-mediated pathway known as break-induced replication (BIR) that serves to re-establish the replication fork and allow completion of DNA synthesis. Because of the aforementioned evidence that WRN might function in HR-mediated repair of DSBs, an accessory role for WRN in BIR could also be envisioned. Such a role would be consistent with the chromosomal rearrangements observed in WRN-deficient cells [111] and their hypersensitivities to agents that could cause blockage or collapse of replication forks [117] as well as the relocalization of WRN to replication forks after treatment with these agents [105]. However, additional evidence is needed to support the involvement of WRN in BIR pathways. In cells treated with high concentrations of CPT (or prolonged treatment with HU), it is known that replication forks collapse to form DSBs. In a reaction that requires NBS1, WRN protects the nascent DNA strands from degradation by MRE11 [133]. Surprisingly, neither the exonuclease nor helicase activities of WRN are required for this function [133]. In the absence of WRN, MUS81 nuclease (or ectopically expressed bacterial resolvase RusA) has been shown to resolve structures that form and allow subsequent HR reactions that involve RAD51 [113,154].

### 5.7. Okazaki Fragment Maturation and Repair

Another potential replication-related role for WRN that has been promoted is in aiding synthesis and maturation of Okazaki fragments. Okazaki fragments are created during discontinuous lagging strand synthesis; their “maturation” involves removing the RNA primer section of the adjacent fragment, resynthesizing those segments using deoxynucleotides, and, finally, ligation to yield a continuous lagging daughter strand. A role for WRN in the completion of lagging strand synthesis has been supported mainly by its functional interactions with DNA polymerase δ (pol δ), FEN1, and PCNA. Pol δ is responsible for lagging strand synthesis after each Okazaki fragment is initially primed by DNA polymerase *α*, and PCNA increases the processivity of pol δ. FEN1 cleaves 5′ flaps (containing the RNA primer from an adjacent Okazaki fragment) generated by displacement synthesis by pol δ. The direct interaction of WRN with pol δ increases the latter’s processivity, fidelity, and ability to synthesize through regions that form secondary structures, in a manner that may also involve PCNA [155,156,157,158]. WRN interacts directly with FEN1 and stimulates its activity in cleaving 5′ flap structures, possibly in coordination with PCNA [58,159,160]. Notably, BLM could also stimulate FEN1 cleavage activity [161]. Finally, in an elegant series of biochemical experiments, Comai and colleagues [158] demonstrated that, in a single assay, WRN could stimulate both (1) pol δ synthesis through a region containing G-rich telomeric sequences known to form a G-quadruplex structure and (2) FEN1-mediated cleavage of the RNA primer section of a duplex modeled to represent an adjacent Okazaki fragment. In this system, BLM could stimulate pol δ synthesis but could not stimulate FEN1 cleavage [158]. Taken together, these data promote a role for WRN in lagging strand synthesis that would not only help pol δ overcome secondary structures that might form in the parental lagging strand but also coordinate lagging strand synthesis with FEN1-mediated removal of RNA primers from the adjacent Okazaki fragment. Thus, WRN deficiency might be expected to cause problems with (lagging strand) replication, including persistence of single-stranded gaps that might be subject to cleavage, resulting in collapse of replication forks. Although WRN might perform such a function across the genome, it might explain the lagging strand telomere deletion phenotype associated with loss of WRN function (see below). Although biochemical studies are highly supportive, more in vivo evidence is needed to confirm these roles for WRN in Okazaki fragment maturation.

### 5.8. Telomere Maintenance

Early studies by George Martin and colleagues, as well as by other labs, demonstrated that primary fibroblasts derived from skin biopsies of WS patients underwent very rapid cellular senescence, generally prior to 30 population doublings as compared to 40–100 population doublings in fibroblasts from normal individuals [110,162,163]. After the link between telomere shortening and cellular senescence was established in the 1990s, research zeroed in on a possible deficit in telomere maintenance in WS. Consistent with this hypothesis, transfection of the *TERT* gene encoding the catalytic protein subunit of telomerase into primary WS fibroblasts resulted in their immortalization [164], pointing to telomere length issues in cells lacking WRN function. However, mean telomere lengths measured in cell populations did not appear to shorten faster in WS fibroblasts compared to normal fibroblasts [165]. Instead, subsequent studies showed that cells lacking WRN function had occasional stochastic telomere deletions on isolated chromosomes while telomeres on the remaining chromosomes appeared to be normal [48,166]. One of these studies indicated that these telomeric deletions were occurring in relation to lagging strand replication [48]. Importantly, drastically shortened telomeres due to deletions lose their protected state, and unprotected telomeres trigger ATM-mediated checkpoint activation that can result in cell death or cellular senescence [167,168]. One can speculate about several functions for WRN in telomere maintenance that somewhat parallel its roles in the remainder of the genome (Figure 2B).

These cellular studies have been supported by mouse models of WS, but not without substantial consternation as mice that only lacked WRN function grew and aged in a relatively normal manner [169,170,171]. However, creation of more complex WRN-deficient models was spurred by two observations: (1) lab mice have extremely long telomeres (20–50 kb) compared to humans (2–15 kb) and (2) even the TERT (telomerase) knockout mouse did not show phenotypes until the sixth generation [172,173]. Importantly, a mouse knockout of WRN that was superimposed on telomerase deficiency recapitulated at least some of the aging features of WS, but these did not appear unless telomeres were “preshortened” in the fourth generation of telomerase deficiency [174,175]. Notably, cells isolated from these WRN- and TERT-deficient mice also showed the stochastic telomere deletion phenotype mentioned above [176]. One of these groups [174] made a triple knockout that lacked BLM, WRN, and TERT function; in this mouse, premature aging characteristics were apparent even in the second generation of telomerase deficiency. This suggests that BLM also has a telomere maintenance function that is either independent of or somewhat redundant to WRN function. 

Based on this evidence, it seems reasonably clear that at least part of the excess cell death and/or cellular senescence in WS is related not to accelerated universal shortening at every telomere but instead to apparently random large deletions occurring at a small subset of the telomeres in an individual cell. The presence of sufficient telomerase activity in a cell would be able to add back telomeric sequences to these deleted telomeres and thus rescue the cell from checkpoint activation and cell senescence (or death). It can be speculated that telomerase and WRN participate in distinct pathways that help maintain telomere length and function. When telomerase activity in a cell is low or absent, loss of WRN function has serious consequences for telomere maintenance and cell fate. When telomerase activity is present, WRN function becomes less important or perhaps even dispensable. 

WRN has a preference for binding to and acting on DNA substrates containing telomeric sequences especially when placed in a particular structural context [54]. Intriguingly, this structural context is relevant to recombinational strand invasion intermediates, including the protective T-loop structures that form at telomeres, and WRN action on such structures tends to promote further strand invasion [54]. WRN has also been shown to have interactions with several key telomeric/shelterin factors including POT1, TRF1, and TRF2 [65,103,177,178]; POT1 specifically binds single-stranded G-rich telomeric sequences, while both TRF1 and TRF2 bind double-stranded telomeric sequences. The aforementioned WRN–Ku interaction should also be noted in this context, as Ku is also partly localized at telomeres. Of these, the WRN–TRF2 interaction would appear to be the most functionally important. WRN and TRF2 directly bind to each other even in the absence of DNA, co-immunoprecipitate from cell lysates, and colocalize in nuclear foci [103,178]. This interaction appears to require the N-terminal region of TRF2 [179]. TRF2 also influences the enzymatic activities of WRN and mostly in a positive or stimulatory manner. Specifically, TRF2 stimulates both the helicase and exonuclease activities of WRN on DNA substrates, particularly those containing telomeric sequences [90,103,178]. Notably, the helicase activity of BLM on telomeric substrates is also stimulated by TRF2 [178]. Moreover, TRF2 (but not TRF1) can specifically stimulate WRN-mediated strand exchange between substrates containing duplex telomeric regions, suggesting that WRN and TRF2 might act together during telomeric recombination processes [90]. Whatever the function of WRN in telomere maintenance, TRF2 appears to help recruit WRN to duplex telomeric DNA to carry out that function. Cooperation of WRN with TRF1 and POT1 has also been reported [65,177], and these interactions may serve to further regulate the telomere maintenance function of WRN.

What is the specific function of WRN in telomere metabolism? A number of studies have shed more light on this. WRN association with telomeric DNA becomes enriched during S phase [48,65]. The evidence indicates that WRN’s role in telomere maintenance requires its helicase activity and suppresses the appearance and/or accumulation of DNA breaks in telomeres in relation to replication through telomeric regions [48,180], possibly in coordination with TRF2 and other telomeric factors. It should be noted that telomeres are “fragile sites”, i.e., sequences in the genome at which double-strand breaks occur in a replication-related manner and happen at a higher than random frequency [181]. Based on the stochastic telomere deletion phenotype of WRN-deficient cells, two possibilities come to mind: (1) WRN directly prevents DNA break accumulation in telomeres or (2) WRN serves to rescue breaks that occur in telomeric regions, thus reducing their persistence. In both cases, the downstream effects of telomere loss and dysfunction, i.e., checkpoint activation leading to either cellular senescence or apoptosis or telomere-related chromosomal instability, are avoided. One possibility is that WRN functions to facilitate replication through telomeric regions; when WRN (helicase and/or exonuclease) function is lacking, telomeric replication might become stalled or blocked, directly deleting one or both sister chromatids at that site. The observation that WRN deficiency results in occasional deletions related to lagging strand replication [48] points to a specific problem pertaining to replication of the telomeric G-rich strand, which is almost always the lagging strand due to origins generally being positioned centromeric to telomeric regions. Since the parental single-stranded G-rich telomeric sequences revealed during lagging strand replication might be prone to form (intramolecular) G-quadruplex structures, it was suggested that WRN might help disrupt G-quadruplexes [48]. Indeed, WRN can disrupt G-quadruplexes formed from telomeric sequences, at least in biochemical reactions [63]. In the absence of WRN, these structures and nearby single-stranded parental DNA would persist and possibly be subject to cleavage by nucleases, resulting in deletion of the lagging strand telomere distal to that point that would be consistent with the observation of stochastic telomeric deletions in WRN-deficient cells. Notably, cells deficient in WRN, BLM, or RECQ4 have transcriptional alterations related to sequences with the potential to form G-quadruplexes [182].

Another possibility is that WRN is involved in metabolism of T-loops (telomeric D-loops), a structure in which single-stranded G-rich 3′ overhangs are inserted into the duplex telomeric region. This structure serves to sequester telomeric ends to avoid checkpoint activation, telomere–telomere fusions, and their degradation [183] but must be resolved prior to passage of replication forks and be re-established after telomeric replication and related processing events are completed. As mentioned above, D-loop structures are high-affinity substrates of WRN [57]. Furthermore, T-loop-like structures with G-rich telomeric sequences on the invading strand (its precise context in T-loops) are even more preferential substrates for the DNA-binding and unwinding activities of WRN [54]. Helicase directionality experiments on these substrates indicate that, at physiological salt concentrations, WRN preferentially acts in a manner that would promote the formation of T-loops [54]. Conversely, other biochemical experiments suggest that WRN might act to resolve T-loop structures [65]. Although further experiments are needed to distinguish between these possibilities, the role for WRN function in T-loop dynamics is consistent with many of the telomeric phenotypes observed in WRN-deficient cells. 

Last but not least, WRN may act in a recombinational pathway that rescues collapsed replication forks in telomeres; since telomeres are fragile sites, such events might not be that rare. The ability of G-rich telomeric sequences to form G-quadruplexes might contribute to this fragility. A telomere recombinational role for WRN is consistent with the role of its *S. cerevisiae* homolog Sgs1 in telomere maintenance [184] and the observation that, in cells using the alternative lengthening of telomeres (ALT) pathway, WRN and BLM are associated with telomeric DNA and with PML bodies that contain telomeric and recombination factors [65,184,185]. Furthermore, TRF2 (but not TRF1) promotes WRN helicase-dependent strand exchange between telomeric DNA substrates [186], a finding that supports coordination between these factors to facilitate telomeric recombination processes. The idea that WRN might also be involved in resection of one-ended breaks resulting from collapsed forks [49] could also support a function in telomeric recombination. Together, these findings suggest that, following replication fork collapse in telomeric regions, WRN may act in a BIR homologous recombination pathway to promote replication restart and rescue of broken telomeres [186]. Such a role would again be consistent with the stochastic telomere deletion phenotype observed when WRN function is absent.

## 6. Post-Translational Modification and Regulation of WRN

As alluded to above, WRN is subject to several post-translational modifications, including phosphorylation, acetylation, ubiquitination, and SUMOylation. These modifications have been documented mainly in response to cellular perturbations (primarily DNA-damaging treatments), and some modifications have been shown to influence WRN’s cellular localization, enzymatic activities, and physiological functions. Nonetheless, it remains to be elucidated precisely how specific modifications regulate the physiological functions of WRN. 

Important and timely post-translational phosphorylation of WRN is accomplished by some phosphoinositol-3 kinase-related kinase (PIKK) kinases (namely ATR, ATM, and DNA-PKcs) and by cyclin-dependent kinases. The PIKK kinases phosphorylate SQ/TQ motifs in target proteins; there are six such sequences (Figure 1) in the C-terminal region (between aa991 and aa1292) of WRN [150]. ATM and ATR oversee checkpoints responding to double-strand breaks and replication stress, respectively, while DNA-PKcs is a central factor in the NHEJ pathway for the repair of double-strand breaks. Early studies indicated that WRN could be phosphorylated by DNA-PKcs in cells as well as in vitro, suggesting a possible role in NHEJ [101,102]. Although the findings of these studies are not completely consistent, both show a direct interaction between DNA-PKcs and WRN, the formation of quaternary complexes containing WRN, Ku, DNA-PKcs, and DNA, and that WRN phosphorylation was reduced in cells lacking DNA-PKcs or in cells treated with wortmannin, which preferentially inhibits DNA-PKcs compared to other PIKK kinases [101,102]. WRN phosphorylation was increased by treatment with bleomycin or 4NQO, oxidative DNA-damaging agents that can induce DSBs [101]. The results from both papers also suggested that DNA-PKcs-mediated phosphorylation of WRN inhibited its exonuclease activity and possibly its helicase activity [101,102]. Taken together, these studies suggest that DNA-PKcs-mediated phosphorylation of WRN might regulate the latter’s function as an accessory factor in NHEJ. 

In response to replication stalling caused by treatment with HU, CPT, or even UV-C light, WRN is phosphorylated in its C-terminal region (at S991, T1152, and/or S1256) during S phase in an ATR-dependent manner and possibly at other sites by ATM [115,150]. These phosphorylation events appear not to affect ATR’s cell cycle arrest function but do appear to influence WRN localization at nuclear foci reflecting blocked replication, although they are not absolutely required for initial relocation to these foci [115,150]. Perhaps more importantly, mutation of the ATR phosphorylation sites in WRN enhanced the formation of DSBs in cells after treatment with HU or CPT, suggesting that ATR-dependent WRN phosphorylation is important for replication fork stabilization and delaying or preventing fork collapse [150]. Interestingly, expression of a WRN mutant that could not be phosphorylated by either ATR or ATM appeared to have a more deleterious effect after replication stalling than complete lack of WRN, indicating a dominant-negative effect possibly caused by interference with a RAD51-mediated HR pathway for repair of collapsed forks [150]. Notably, ATM-mediated phosphorylation of WRN (putatively at S1108, S1141, and/or S1292) has been suggested to be linked to DSB formation caused by fork collapse and may be involved in allowing an HR pathway to take over after initial fork repair efforts fail [115,150]. In general terms, the evidence suggests that WRN phosphorylation by ATR and ATM is important for regulating pathways invoked following replication stress that mediate replication fork recovery and thereby prevent genome instability and cell death.

Phosphorylation of WRN by ATR at S1141 appears critical for WRN’s response to replication stress and eventually paves the way for WRN ubiquitination and degradation [187]. More recently, a separate phosphorylation of WRN at S1133 dependent on CDK1 has been reported [50]. Importantly, this phosphorylation event appears to mediate WRN interaction with the MRN (MRE11/RAD50/NBS1) complex that is critical in the checkpoint and repair responses to DSBs [50,132,188]. Evidence indicates that this CDK1-dependent phosphorylation promotes a function for WRN in DNA2-catalyzed long-range resection to facilitate HR-mediated repair of (one-ended) DSBs produced by CPT treatment, and expression of a WRN mutant (S1133A) that cannot be phosphorylated by CDK1 causes chromosomal instability and cell death [50]. These findings support a role for WRN in the resection step of HR repair of one-ended DSBs that result from replication fork collapse.

In addition to phosphorylation, WRN acetylation has also been reported. Acetylation of WRN is observed in response to treatment of cells with mitomycin C, methylmethanesulfonate, and other DNA-damaging agents [105,106,107,108]. p300 acetyltransferase appears to be responsible for WRN acetylation, while SIRT1 mediates its deacetylation [189]. Interestingly, acetylation of WRN correlates well with its translocation to nuclear foci (from either a nucleolar or diffuse nuclear localization), suggesting a role in its recruitment to sites of blocked replication [106,107]. WRN acetylation may also increase its stability by inhibiting ubiquitination and proteasomal degradation [190]. Furthermore, acetylation of WRN modifies its DNA-binding specificity and thus its unwinding and exonuclease activities [108]. It has been speculated that WRN acetylation reduces non-specific DNA binding and thereby increases its specificity for its physiological DNA targets [108]. 

Phosphorylation of WRN by ATR or ATM has been shown to lead to its ubiquitination [187,191]. Under different conditions, WRN can be ubiquitinated by E3 ubiquitin ligases MDM2 and MIB1; under either circumstance, WRN ubiquitination leads to its degradation [192,193]. Additionally, WRN is reported to be subject to SUMOylation, i.e., conjugation with small ubiquitin-like modifying protein. Initially, interactions among the mouse homologs Wrn, Ubc9, and SUMO-1 were identified using a yeast two-hybrid system [194]. Subsequently, ectopically expressed WRN homologs in mouse and human cells were shown to be modified specifically by SUMO-1 in a Ubc9/UBCH9-dependent manner [194,195]. Multiple putative SUMOylation sites in human WRN were mapped to K356, K370, K496, and K898, and even more sites towards the C-terminus are suggested [195]. Interestingly, the tumor suppressor p14ARF stimulated WRN SUMOylation, and this modification was linked to the exclusion of WRN from the nucleolus [195]. Potential ubiquitination and SUMO modifications of WRN and their physiological function need more study.

## 7. Synthetic Lethal Interactions

WRN has been shown to have what appears to be synthetic lethal interactions with other proteins and pathways. WRN loss or depletion caused recruitment and action of MUS81 in the presence of replication stress and further depletion of MUS81 in WRN-deficient cells has been associated with increased cytotoxicity [154]. After treatment with CPT, HU, or other agents that stall replication, cells lacking WRN become dependent on RAD51 and HR pathways for recovery [47,154]. Thus, it seems likely that WRN has a synthetic lethal interaction with RAD51 and perhaps other key recombination factors, although deletion of RAD51 on its own makes replicating cells very unhealthy due to the critical roles of HR in cell viability. WRN inhibitors sensitize cancer cells to topoisomerase inhibitors and to mitomycin C in FA cells that have defects in repair of interstrand crosslinks, suggesting that WRN may play a role in the repair of these types of lesions [196,197]. WRN inhibitor also sensitized cancer cells to the G4 stabilizer telomestatin [196], consistent with its role in telomere maintenance. 

Recently, WRN has been shown to have a synthetic lethal interaction with microsatellite unstable colon cancer lines, suggesting possible synthetic lethal interactions with mismatch repair (MMR) [198,199]. The microsatellite unstable cancer cell lines showed a massive increase in double-strand breaks when WRN was not functional, leading to a substantial increase in cell death [198,199]. Interestingly, this was not due to a synthetic lethal interaction with MMR proteins but to TA dinucleotide repeat sequences that had rapidly expanded in microsatellite unstable cancers and formed secondary DNA structures that inhibited replication and were thus dependent upon WRN for their resolution [200]. Thus, downregulating WRN function may be a viable strategy in microsatellite unstable cancers, but this would depend upon their having many expanded TA repeat sequences in those cancers. It should be feasible to find that subset of microsatellite unstable cancers with numerous TA dinucleotide expansions that would be susceptible to killing by a WRN-specific inhibitor.

## 8. Conclusions

Since the identification of the *WRN* gene responsible for WS in 1996, research has shed much light on the defects caused by loss of WRN function. However, we still are unclear about the precise roles for WRN in DNA metabolism. Most studies point to WRN action in resolving DNA structures that inhibit replication or in mediating reactions on DNA intermediates downstream of stalling or blockage of replication (Figure 2A). WRN’s helicase activity and perhaps its strand exchange activity are required for most of these reactions; WRN’s exonuclease activity appears to be required for some processes but not others. WRN functions in telomere maintenance, but it is unclear whether its role is particularly specialized for telomeric structures or whether its normal function in facilitating replication is particularly important in telomeric regions (Figure 2B). Regardless, in normal cells, loss of WRN function causes partially redundant and error-prone backup pathways to kick in; this can lead to chromosomal aberrations and telomeric attrition, the latter of which definitely causes increased apoptosis and cellular senescence. The increased chromosome aberrations lead to earlier cancers in WS patients. Accumulation of senescent cells leads to earlier onset of the senescence-associated secretory phenotype or loss of cell proliferation capability in particular tissues, thus leading to the segmental progeroid phenotypes observed in WS.

## Data Availability

No new data were created or analyzed in this study. Data sharing is not applicable to this article.

## Abbreviations

BS = Bloom syndrome; CPT = camptothecin; DNA-PKcs = DNA-dependent protein kinase catalytic subunit; DSB = double-strand break; HRDC = helicase and Rnase D-conserved; HJ = Holliday junction; HR = homologous recombination; HU = hydroxyurea; NHEJ = non-homologous end joining; NLS = nuclear localization signal; RQC = RecQ Conserved; RTS = Rothmund–Thomson syndrome; SASP = senescence-associated secretory phenotype; WS = Werner syndrome.
